# A retrospective study of clinical and laboratory features and treatment on cats highly suspected of feline infectious peritonitis in Wuhan, China

**DOI:** 10.1038/s41598-021-84754-0

**Published:** 2021-03-04

**Authors:** Yiya Yin, Ting Li, Chaohao Wang, Xiaoya Liu, Hehao Ouyang, Wanfeng Ji, Jiahao Liu, Xueyu Liao, Junyi Li, Changmin Hu

**Affiliations:** College of Veterinary Medicine, Huazhong Agricultural University, Rm 321 Vet. Teaching Hospital BLDG, No.1 Shizishan St., Hongshan District, Wuhan City, 430070 Hubei Province China

**Keywords:** Zoology, Infectious-disease diagnostics

## Abstract

Feline infectious peritonitis (FIP) is a systemic, potentially fatal viral disease. The objectives of this study were to review clinical and laboratory features and treatment of cats highly suspected of FIP in Wuhan, China. The clinical records of 127 cats highly suspected of FIP were reviewed for history, clinical signs, physical findings, and diagnostic test results. Sex, neutering status, breed, age, and month of onset of disease were compared with the characteristics of the clinic population. Age and neutering status were significantly correlated with FIP-suspicion. Sex, breed and onset month were not associated with FIP. There were many more FIP-suspected cases in cats in young cats or male intact cats. Effusion was observed in 85.8% of the FIP-suspected cats. Increased serum amyloid A (SAA) and lymphopenia were common laboratory abnormalities in the FIP cases. Furthermore, 91.7% of the cats highly suspected of FIP had an albumin/globulin (A/G) ratio < 0.6, while 85.3% had an A/G ratio < 0.5. The mortality rate for FIP-suspected cats was 67%, and six submitted cases were confirmed by FIP-specific immunohistochemistry. Of the 30 cats treated with GS-441524 and/or GC376, 29 were clinically cured. The study highlights the diverse range of clinical manifestations by clinicians in diagnosing this potentially fatal disease. A/G ratio and SAA were of higher diagnostic value. GS-441524 and GC376 were efficient for the treatment of FIP-suspected cats.

## Introduction

Feline infectious peritonitis (FIP) is a worldwide disease of domestic and wild felids. The pathogen of FIP is feline infectious peritonitis virus (FIPV), which is mutated from feline coronavirus (FCoV)^1,2^. Although much research into FIP has been performed, FIP remains one of the most prevalent and fatal infectious diseases of cats.

Except for non-specific clinical signs, such as lassitude, inappetence, fluctuating fever and weight loss, the clinical presentation of FIP is complex and variable. Different clinical features may be a result of variations in the virus and in the nature of the individual host’s immune response. FIP can be divided into three types—effusive, non-effusive and mixed—according to the different clinical features that emerge, but these types can change over time in any individual cat. Effusive FIP is mainly manifested as ascites and/or pleural effusion, while non-effusive FIP is mainly manifested as granuloma formation involving the central nervous system, eyes and abdominal organs, and it does not produce body cavity effusion^3^. When effusive and non-effusive signs occur together, it is mixed FIP^4^. To our knowledge, there are few studies about the clinical and laboratory features of FIP in China.

Ante-mortem diagnosis of FIP is a controversial and complicated issue. It is more difficult to diagnose FIP definitively in vivo in cats without effusion as clinical signs and laboratory features are relatively vague. Immunofluorescence staining of FCoV antigen in macrophages is highly specific, but it is difficult to detected due to the low numbers of macrophages in the effusion. At present, the gold standard for the diagnostic of FIP is immunohistochemistry (IHC) to identify FCoV antigens in diseased tissues. However, this invasive method has low operability and requires professional detection equipment and personnel. Also, a negative IHC result does not exclude the diagnosis of FIP, adding to the challenges of diagnosing this disease^5^. FIP has an extremely high mortality rate and was once considered a terminal disease. In recent years, 3C-like protease inhibitor (GC376) and nucleoside analogue (GS-441524) have been proven to have a certain therapeutic effect on FIP^6–9^. The purpose of this study is to determine the epidemiological features and related risk factors of FIP, summarise common clinical signs and laboratory features, and evaluate the therapeutic effects of GS-441524 and GC376.

## Materials and methods

### Selection of cases

This study retrospectively evaluated the medical records in the database of the 12 veterinary hospitals in Wuhan, China, from 1 January 2019 to 1 January 2020. Due to the limitations of the diagnostic facilities in veterinary hospitals and the cat owners were reluctant to consent to invasive diagnostic procedures such as biopsies, most of the cases were not confirmed by IHC. Therefore the following inclusion criteria for the diagnosis of suspected FIP cases were established based on the previously published literature in this field^5–15^ Those cases meeting at least four of the following characteristics were defined as highly suspected of FIP: 1) Typical clinical signs of effusive FIP (pleural effusion and/or ascites) or typical clinical signs of non-effusive FIP (ocular and neurological signs); 2) A decreased albumin/globulin (A/G) ratio of the cut-off (0.6); 3) Rivalta’s test showing positive results in effusion; 4) Positive reverse transcription–polymerase chain reaction (RT-PCR) detection of FCoV RNA in effusion; 5) Tissue samples collected by autopsy showing typical histological features of FIP, namely systemic vasculitis and pyogranulomatous lesions.

Out of 20 984 cats registered in the 12 veterinary hospitals, 127 cases with a high suspicion of FIP were included according to the criteria above. Through basic information collection, history investigation, clinical signs, haematological and biochemical analyses, diagnostic imaging, RT-PCR, Rivalta’s test, exploratory laparotomy, and histopathology, the cases were comprehensively diagnosed and analysed, and detailed information and examination results were recorded (see Supplementary Table S1 for the FIP case record sheet). All information was collected with the consent and cooperation of the veterinarians and cat owners. The protocol was approved by the Animal Management and Ethics Committee of the Laboratory Animal Centre, Huazhong Agriculture University (approval no.: HZAUCA-2018–005).

### Statistical analysis

Statistical analysis was performed using commercial software (SPSS Version 21.0 [IBM]). Descriptive statistics were employed for all evaluated variables. Categorical data were analysed using a Pearson chi-square (χ^2^) test. In 2 × 2 contingency tables with any expected cell values < 5, Fisher’s exact two-tailed results were used. Sex, neutering status, breed, age and month of onset of highly suspected of FIP were compared with the feline clinic population (20 984 cats) presenting to the 12 veterinary hospitals from January 2019 to January 2020 to determine whether these risk factors were associated with FIP.

The history, clinical signs and laboratory examination results of FIP-suspected cases were retrospectively analysed to summarise the common clinical features and laboratory examination abnormalities of the disease. Samples were randomly selected from the cats undergoing exploratory laparotomy and sent to LABOKLIN GMBH & CO. KG (Bad Kissingen, Germany) for IHC to verify whether the inclusion criteria for FIP cases were correct and reasonable. In addition, the therapeutic effect of GS-441524 and GC376 was evaluated.

### Ethical approval

All applicable international, national and institutional guidelines for the care and use of animals were followed. All procedures performed in studies involving animals were in accordance with the ethical standards of the institution at which the studies were conducted.

## Results

### Risk factors

There was no significant correlation between FIP and sex (*p* = 0.083). However, FIP was significantly correlated with neutering status (*p* < 0.001), and male intact cats were more susceptible to disease. As for breed, whether classified as crossbred or purebred (*p* = 0.069) or specific to each breed (*p* = 0.246), no correlation with the FIP was found. There was a significant correlation between age and FIP (*p* < 0.001), and young cats were more susceptible. The age of the FIP-suspected cases ranged from 1 month to 8 years and 2 months, with an average age of 13.1 months and a median age of 8 months. Within these cases, 40.2% were under 6 months old, 67% were under 1 year old and 90.6% were under 2 years old; moreover, the prevalence of older cats was significantly lower than that of young cats (Table 1). The correlation of onset month was studied for the first time, and the number of FIP-suspected cats and clinic population in each month was counted. The results showed that the highest prevalence was 1% in May. The prevalence in January (0.95%), February (0.87%), September (0.66%) and November (0.77%) were higher than the total prevalence, and the lowest prevalence was 0.35% in July. However, in the statistical analysis, there was no correlation between FIP and month (*p* = 0.135; Table 1).

**Table 1:** Risk factors of cats highly suspected of FIP compared with the clinic population.

Risk factors	FIP-suspected population (*n* = 127)	Clinic population (*n* = 20,984)	χ^2^	*p* value	OR	95% CI
**Sex**	*n* = 127 (%)	*n* = 17,494 (%)	3.009	0.083		
Male	78 (61.4)	9397 (53.7)			1.372	0.958–1.963
Female	49 (38.6)	8097 (46.3)			0.729	0.509–1.043
**Neutering status**	*n* = 127 (%)	*n* = 16,041 (%)	45.684	< 0.001***		
Intact male	69 (54.3)	4887 (30.5)			Ref	
Neutered male	9 (7.1)	3791 (23.6)			0.168	0.084–0.337
Intact female	39 (30.7)	4472 (27.9)			0.618	0.416–0.917
Spayed female	10 (7.9)	2891 (18.0)			0.245	0.126–0.476
**Breed**	*n* = 127 (%)	*n* = 16,469 (%)	3.307	0.069		
Crossbred	36 (28.3)	5953 (36.1)			0.699	0.474–1.029
Purebred	91 (71.7)	10,516 (63.9)			1.431	0.972–2.108
**Breed (specific)**	*n* = 127 (%)	*n* = 16,469 (%)	16.752	0.053		
Crossbred	36 (28.3)	5953 (36.1)			Ref	
British Shorthair	60 (47.2)	6558 (39.8)			1.513	0.999–2.290
American Shorthair	8 (6.3)	1275 (7.7)			1.038	0.481–2.237
Ragdoll	9 (7.1)	877 (5.3)			1.697	0.815–3.535
Siamese	6 (4.7)	430 (2.6)			2.307	0.967–5.506
Exotic Shorthair	3 (2.4)	1019 (6.2)			0.487	0.150–1.584
Chinchilla	2 (1.6)	258 (1.6)			1.282	0.307–5.353
Longhair Scottish Fold	1 (0.8)	44 (0.3)			3.758	0.504–28.021
American Curl	1 (0.8)	28 (0.2)			5.906	0.782–44.581
Bengal	1 (0.8)	27 (0.2)			6.124	0.810–46.291
**Age groups**	*n* = 127 (%)	*n* = 19,772 (%)	220.276	< 0.001***		
≤ 6 M	51 (40.2)	1530 (7.7)			Ref	
6 M < A ≤ 1Y	34 (26.8)	3254 (16.5)			0.313	0.202–0.486
1Y < A ≤ 2Y	30 (23.6)	4660 (23.6)			0.193	0.123–0.304
2Y < A ≤ 4Y	8 (6.3)	4027 (20.4)			0.060	0.028–0.126
4Y < A ≤ 7Y	3 (2.4)	3507 (17.7)			0.026	0.008–0.082
A > 7Y	1 (0.8)	2794 (14.1)			0.011	0.001–0.078
**Onset months**	*n* = 127 (%)	*n* = 20,984 (%)	16.166	0.135		
January	16 (12.6)	1685 (8.0)			Ref	
February	13 (10.2)	1500 (7.1)			0.913	0.438–1.904
March	7 (5.5)	1768 (8.4)			0.417	0.171–1.016
April	7 (5.5)	1656 (7.9)			0.445	0.183–1.085
May	17 (13.4)	1689 (8.1)			1.054	0.531–2.094
June	10 (7.9)	2109 (10.1)			0.499	0.226–1.103
July	7 (5.5)	2022 (9.6)			0.365	0.150–0.888
August	9 (7.1)	1773 (8.4)			0.535	0.236–1.213
September	12 (9.4)	1812 (8.6)			0.697	0.329–1.479
October	8 (6.3)	1734 (8.3)			0.486	0.207–1.138
November	13 (10.2)	1698 (8.1)			0.806	0.387–1.681
December	8 (6.3)	1529 (7.3)			0.551	0.235–1.291

Data were given as *n* (%); χ^2^ = Chi-square; OR = Odds Ratio; CI = Confidence Interval; Ref = Reference Category; A = Age; Y = Years; ***: *p* < 0.001, indicating a statistically significant difference.

**Figure 1:**
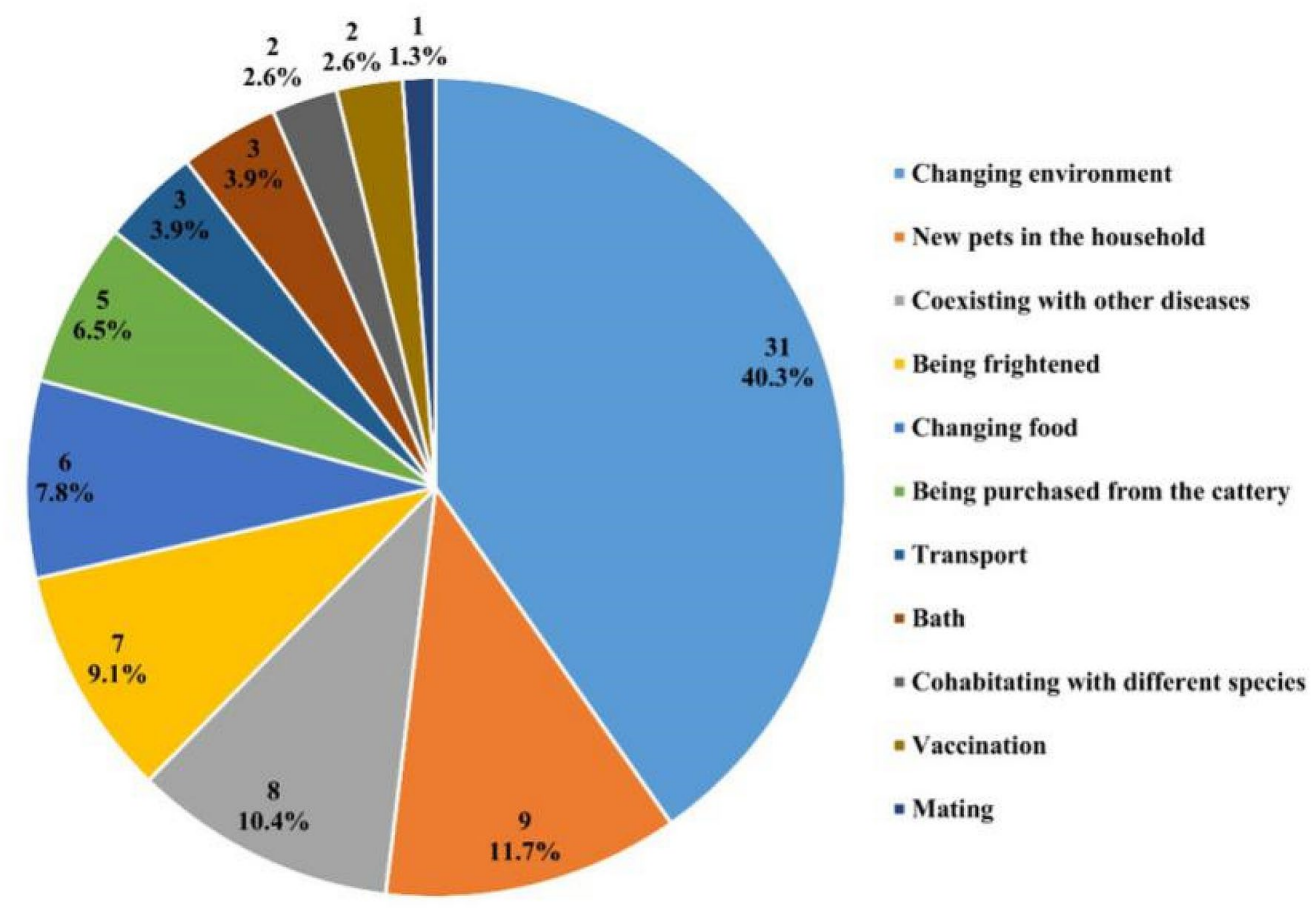
Frequency of documented stress situation of cats highly suspected of FIP.

Stressful events before the diagnosis were documented for 60/127 cats. Since some cats had multiple stressors, a total of 77 stressors were recorded. Of these events, changing environment was the most common factor leading to stress (40.3%), followed by new pets in the household, coexisting with other diseases, being frightened, changing food (Fig. 1). In addition, information on the housing density of 83 cats was collected, of which 43 (51.8%) were single-cat households and 40 (48.2%) were multi-cat households.

### Clinical signs

Of the 127 cats highly suspected of FIP, 12 (9.4%) presented with non-effusive FIP and 109 (85.8%) presented with effusive FIP, of which 92 presented with ascites (84.4%), 11 with pleural effusion (10.1%), and 6 with both ascites and pleural effusion (5.5%). In addition, 6 cats (4.7%) presented with mixed FIP. Some presented with non-effusive FIP first, followed by ascites and the development of effusive FIP. Some cats showed effusive FIP first and develop neurological signs a few days before dying.

The clinical signs of cats highly suspected of FIP are shown in Table 2. The most common non-specific signs were weight loss (93.8%), lassitude (86.2%) and inappetence (86%). Icterus and fever were also present in more than half of the cases, 59.4% and 52.9%, respectively. Of the 85 cats highly suspected of FIP for which the temperature was documented on physical examination, 52.9% had a temperature above 39.5℃, 36.5% had a temperature above 40℃ and 10.6% had a temperature above 40.5℃ (see Supplementary Fig. S1). In the results, 41.3% of the cats had a palpable mass on abdominal palpation, which could be either mesenteric lymph node enlargement or accretion of the intestinal wall. Diarrhoea was uncommon, occurring in only 17.6%, while dyspnoea occurred in 35.2%, and was most common in cats with pleural effusion. In addition, ocular and neurological signs generally occurred only in non-effusive or mixed forms of suspected FIP cases.

**Table 2:** Clinical signs of cats highly suspected of FIP.

Clinical signs	Animals examined (*n*)	Number of cats with clinical signs (*n*)	Percentage of cats with clinical signs (%)
Weight loss	64	60	93.8
Lassitude	94	81	86.2
Inappetence	100	86	86
Icterus	64	38	59.4
Fever	85	45	52.9
Abdominal mass	63	26	41.3
Dyspnoea	71	25	35.2
Diarrhoea	85	15	17.6
Ocular signs	103	9	8.7
Neurological signs	103	9	8.7
**FIP types**			
Non-effusive	127	12	9.4
Ascites	127	92	
Effusive Pleural effusion	127	11	85.8
Both	127	6	
Mixed	127	6	4.7

Ocular signs included uveitis, corneal oedema, hyphema, anisocoria, and retinal detachment. Neurological signs included ataxia, paralysis of the hind limbs, nystagmus, twitching, and salivation. Data were given as *n.*

### Haematology and serum biochemistry

As shown in Table 3, haematocrit (HCT) decreased in 40.2% of the cats, but only 15.9% of the cats had a decrease in red blood cell (RBC) count. Half of the cats developed lymphocytopenia, 33.9% had an increase in white blood cells (WBC), and 34.3% had an increased neutrophil (NEU) count.

**Table 3:** Haematology and serum biochemistry of cats highly suspected of FIP.

Measurement	Unit	Reference interval	Animals examined	Range	Mean	Median	Increased	Decreased	Normal
Red blood cells	× 10^12^/L	5–10	107	3.22–12.5	6.78	6.81	7 (6.5)	17 (15.9)	83 (77.6)
Haematocrit	%	24–45	82	13.8–46.6	26.68	26	2 (2.4)	33 (40.2)	47 (57.3)
White blood cells	× 10^9^/L	5–18.9	109	0–71.9	17.23	15.6	37 (33.9)	8 (7.3)	64 (58.7)
Lymphocytes	× 10^9^/L	1.5–7.8	106	0–27.8	3.42	1.45	11 (10.4)	53 (50.0)	42 (39.6)
Neutrophils	× 10^9^/L	2.5–12.5	35	0–30.83	9.7	7.93	12 (34.3)	8 (22.9)	15 (42.9)
Total protein	g/L	57–89	109	47–120	75.77	76	16 (14.7)	11 (10.1)	82 (75.2)
Albumin	g/L	22–40	109	10–33	20.96	21	0	64 (58.7)	45 (41.3)
Globulin	g/L	28–51	109	30–96	55.03	53	63 (57.8)	0	46 (42.2)
Albumin/globulin ratio	/	/	109	0.137–0.767	0.399	0.385	/	/	/
Total bilirubin	µmol/L	0–15	76	0.8–147.1	25.2	15	37 (48.7)	0	39 (51.3)
Alanine aminotransferase	U/L	12–130	101	10–488	49.31	35	5 (5.0)	13 (12.9)	83 (82.2)
Alkaline phosphatase	U/L	14–111	74	0–155	35.53	27.5	3 (4.1)	19 (25.7)	52 (70.3)
Creatinine	µmol/L	71–212	94	7.3–302	79.04	71	1 (1.1)	45 (47.9)	48 (51.1)
Urea	mmol/L	5.7–12.9	89	2.2–44.27	6.05	5.1	2 (2.2)	54 (60.7)	33 (37.1)
Amylase	U/L	500–1500	39	26–3178	1356.01	1187	13 (33.3)	2 (5.1)	24 (61.5)
Lipase	U/L	100–1400	11	81–1163	460.91	470	0	2 (18.2)	9 (81.8)
Feline serum amyloid A	mg/L	0–8	44	16.9–253	121.82	118.35	44 (100.0)	0	0

Data were given as *n* (%).

In serum biochemistry, the total protein (TP) levels were mostly normal (75.2%); a few (14.7%) cats had an increased level and a very few had a decreased level. More than half of the cats highly suspected of FIP showed hypoalbuminemia (58.7%). Although the albumin (ALB) levels of the remaining cats were normal, they were also near to the lower limit of the reference range. Hyperglobulinemia was also common in cats highly suspected of FIP (57.8%), and the remainder of the cats had normal globulin (GLOB) levels. The A/G ratio ranged from 0.137 to 0.767, with an mean of 0.399 and a median of 0.385. Among the results, 91.7% were lower than 0.6, 85.3% were lower than 0.5 and 54.1% were lower than 0.4 (see Supplementary Fig. S2). An increase in total bilirubin (TBIL) was observed in nearly half of the cats highly suspected of FIP (48.7%), and alanine aminotransferase (ALT) and alkaline phosphatase (ALP) were mostly normal. Creatinine (CRE) decreased in 47.9% of the cats, while urea decreased in 60.7% of the cats and rarely increased. Amylase (AMYL) and lipase (LIPA) were mostly negative and only positive in fewer cats. In addition, feline serum amyloid A (SAA) increased in all cats (Table 3).

### Imaging and molecular biology

**Figure 2:**
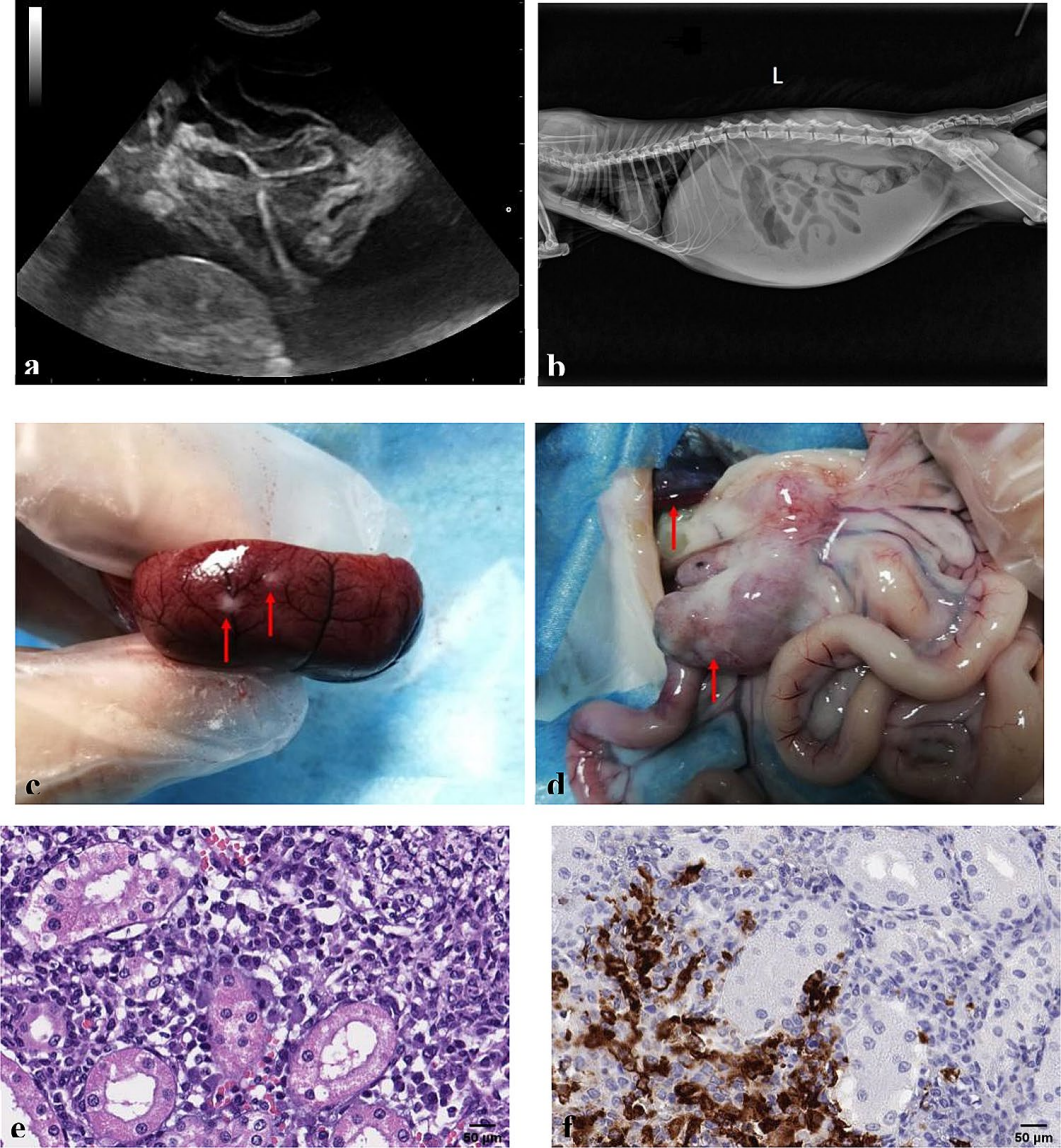
Clinical examination results of a 4-month-old intact female British shorthair cat with effusive FIP. (**a**) The ultrasound showed a large amount of ascites, in which the intestine was floating; (**b**) radiography revealed a large amount of effusion in the abdominal cavity; (**c**) the surface of kidney was scattered with miliary white nodules; (**d**) ascites and enlarged mesenteric lymph nodes were observed. (**e**) Moderate focal pyogranulomatous interstitial nephritis (haematoxylin and eosin stain, × 400). The interstitium of the cortex showed a focal marked infiltration with macrophages, lymphocytes, neutrophils and some plasma cells; (**f**) renal macrophages were stained strongly positive for FCoV antigen with immunohistochemistry, × 400.

Of the 127 cats, 90 were examined by ultrasound, which showed different degrees of ascites, and a few cats demonstrated loss of corticomedullary distinction within the kidneys. Radiography was performed in 21 cats, which showed pleural effusion and/or ascites (Fig. 2). Among 109 cats with effusive disease, 98 had extracted effusion. Then we detected FCoV RNA of 98 effusion by RT-PCR, 83 cats (84.7%) showing FCoV-positive. FCoV mutation was detected in 34 FCoV-positive effusions, with 12 successfully sequenced and partial S gene sequences obtained, all of which had M1058L or S1060A mutations. Furthermore, Rivalta’s tests were performed on 85 cases with a positivity rate of 100%; FCoV antibody tests were carried out in 12 cases, and all were positive. Microscopic examination of smears of the effusions of 25 cats showed many pink protein granules and an increase in inflammatory cells.

### Histopathology and immunohistochemistry

13 cats underwent exploratory laparotomy, and histopathologic diagnosis was performed in 9 cats (see Supplementary Table S2 for details on 13 cats). After exploratory laparotomy, the samples were subjected to histopathological examination and we found the typical pathological changes of FIP, namely systemic vasculitis and pyogranulomatous lesions (Fig. 2). Tissue samples of six cats were randomly selected and sent to LABOKLIN GMBH & CO. KG for IHC. This testing revealed the tissue macrophages were positive-staining for the FCoV antigen, confirming the diagnosis of FIP (see Supplementary Fig. S3–S8).

### Treatment and outcomes

The outcomes of 88 cats were recorded on follow up. Among them, 59 cats eventually died, with a mortality rate of 67%. Thirty-two cats were euthanised directly. Twenty-six cats did not take any treatment or only received treatment for symptoms, and they eventually died naturally. Only one non-effusive FIP cat was treated with GS-441524 for 4 weeks. After remission of the disease, the owner chose to stop the drug, and the cat eventually relapsed and was euthanised. Moreover, 29 cats (25 effusive, 3 non-effusive and 1 mixed) were cured with GS-441524 (2-4 mg/kg, once a day for at least 4 weeks) or GC-376 (6–8 mg/kg, once a day for at least 4 weeks), with a cure rate of 33%. The most obvious signs were rapid reduction of ascites and/or pleural effusion (within 1 week), recovery of mental and appetite and weight gain. The longest survivor was an effusive FIP cat, which was diagnosed on 1 January, 2019 and has survived for more than a year.

## Discussion

In the past, FIP was considered a fatal disease by researchers. Meanwhile, it is difficult to apply the IHC gold standard in the diagnosis of FIP in China. With the development of research, there were gradually found breakthroughs in treatment on FIP, such as studies on GS-441524 and GC376^6–9,16^. But the side effects of these two drugs are not clear, and cannot be used for clinical treatment immediately. Therefore, the establishment of a more accurate diagnostic system is crucial for FIP in China. This paper reviewed 127 FIP cases in Wuhan, China, and studied the diagnostic methods of several hospitals. It emphasizes the clinical manifestations of clinicians in the diagnosis of the disease, and perfects the FIP diagnosis system.

Data on 127 cases highly suspected of FIP were collected in this study, where 13 cats underwent exploratory laparotomy, 9 samples from the cats highly suspected of FIP underwent histopathology, and 6 samples underwent IHC, all of which were finally confirmed as FIP. Therefore, the inclusion criteria for the cases in this study were reasonable. There were also many investigations and studies on FIP based on the analysis of highly suspected cases, which could not be diagnosed by gold standard methods (IHC), but still provided a great reference value for the diagnosis and treatment of FIP^17–22^. In addition, the implementation of invasive diagnostic methods was difficult in clinical practice. Even cat owners who chose euthanasia could not accept autopsy after death. Therefore, when IHC is not available, how to diagnose a FIP-suspected case more accurately is a problem that veterinarians pay close attention to at present.

In this study, the prevalence of cats highly suspected of FIP was 0.61%, and Riemer’s research in Germany showed that the prevalence of FIP was 1.42%^14^. Earlier studies also showed that the prevalence of FIP was 0.02% in single-cat or double-cat households, while the prevalence was 5%–10% in catteries^23,24^. There was no correlation between sex and FIP. However, some studies indicated that male cats were more prone to FIP^25^. The prevalence of intact males was significantly higher than that of other groups, and the prevalence of neutered cats was less, consistent with Rohrbach’s findings^26^. This may be because sex hormones, especially androgens, have a negative effect on the immune system, increasing the risk of virus proliferation and mutation^27^. As reported previously, this study observed that FIP was significantly associated with age, such that young cats were more susceptible, possibly because the immature immune system and various stressors often resulted in high viral loads in young cats. It is possible that the increased rate of unneutered cats may also have been due in part to the young age of infected cats, meaning that the disease occurred before the age of neutering. Purebred cats were previously considered to be more prone to FIP. However, a growing number of studies, including this study, have shown that the proportion of purebred cats was not excessive when compared with the clinic population^14^. This study also explored the regularity of the month of FIP onset, which was not considered in other FIP-related epidemiological surveys, and finally found no significant difference in the prevalence of FIP in each month of the year. This study recorded stressors in 60 cats, the most common of which was changing environment. There was no doubt that stress could inhibit immune function and make viruses more susceptible to mutation and proliferation^10^. Housing density was considered one of the major risk factors for FIP as well, and overcrowding could lead to virus mutation and disease development^14^. However, more of the cats highly suspected of FIP in this study came from single-cat households. This may be because these cats were exposed to FCoV whilst they lived in a multicat household during their infancy, and then moved into single-cat household prior to developing FIP.

Most cases of FIP presented as effusive (up to 85.8%), in which ascites accounted for the majority. FIP could affect systemic organs, most commonly the abdominal organs, including the intestine, mesenteric lymph nodes, liver, kidney, and spleen. Neurological and ocular signs were typical signs of non-effusive FIP. A retrospective study of 286 cats with neurological disease showed that FIP was one of the main causes of neurological disease in cats^28^. In this study, 52.9% of the infected cats had a temperature over 39.5℃, and 36.5% had a temperature over 40℃. Other research has shown that the proportion of cats with fever was larger, where 82% of the cats had a temperature of over 39.5℃ and 39% had a temperature of over 40℃; fever was also found to be more common in effusive FIP^14^. There were no cases of pericardial effusion, scrotal swelling or skin papules, which were not common but could not be ignored in routine examination. The clinical signs of FIP always change over time (such as the gradual production of effusion and changes in the fundus), so repeated clinical examination was highly important to avoid delay in diagnosis and treatment.

Changes in haematology and serum biochemistry could only increase or decrease the suspicion of FIP, but could not be used as a definitive diagnostic method. In this study, there was no obvious indication of hematological changes in the diagnosis of the FIP cases, with HCT decreased in 40.2% of cats, and LYM decreased in 50% of cats. However, many studies have shown that lymphocytopenia is the most common haematological abnormality of FIP induced by natural infection or experiment^29,30^, caused by virus-induced apoptosis of T-cells^31^. In this study, we guess the protein level testing plays an important role in diagnosing FIP cases. ALB decreased in 58.7% of the cats and GLOB increased in 57.8% of the cats, so when both occurred at the same time, TP often remained within the normal range. This was why only 14.7% of the cats showed an increase in TP. Both hyperglobulinemia and hypoalbuminemia could increase the suspicion of FIP, but the most important serum biochemical abnormality was the A/G ratio. The A/G ratio reduction occurred in almost all cases of FIP. Different studies had different results on the effective critical value of the A/G ratio^32^. According to the A/G ratios in this study (91.7% < 0.6, 85.3% < 0.5, and 54.1% < 0.4), the critical value could be considered as 0.5. When the A/G ratio > 0.8, the possibility of FIP was very small; when 0.5 < A/G ratio < 0.8, FIP remained a possibility; when A/G ratio < 0.5, FIP was highly suspected. In addition, FIP was accompanied by inflammatory response, resulting in increased protein concentration in the acute phase protein^33^. Therefore, we guess that the increase in SAA may assist in the diagnosis of FIP.

Compared with serum detection, the detection and analysis of body cavity effusion in cats has better predictive value^32^. The positivity rate of Rivalta’s test was 100% in this study. A study indicated that the sensitivity of Rivalta’s test was 91.3%, the specificity was 65.5%, the positive predictive value was 58.4% and the negative predictive value was 93.4%^34^. Rivalta’s test is a cheap and rapid detection method that does not need expensive instruments and is simple to operate. In view of the good sensitivity and negative predictive value, Rivalta’s test should be included in the diagnosis of every cat with effusion^15^. The positive rate of FCoV RNA detected by RT-PCR was 84.7%. However, all coronaviruses, including FCoV, often mutate and recombine, so RT-PCR designed for specific sequences could not amplify all FCoVs^35^, and the positive results only represented that the cat carried FCoV, not the exact FIPV.

In this study a total of 24 cats were treated with GS-441524, of which 23 were cured. Only 1 cat relapsed after 4 weeks of treatment and was eventually euthanised. Treatment with GS-441524 for more than 8 weeks was highly effective. Treatment with GC376 alone or in combination with GS-441524 was also effective. In fact, most of the final deaths were due to no treatment, symptomatic treatment alone or euthanasia. In addition, Pruijssers and Denison indicated that a combination of potent, broad-spectrum anti-coronavirus drugs can enhance efficacy and reduce the emergence of drug resistance^36^.

In conclusion, FIP was not correlated with sex, breed or month, but it was significantly correlated with age and neutering status. FIP occurred significantly more often in young cats or male intact cats. Effusive FIP was the most common clinical feature. A decreased A/G ratio, increased SAA and lymphopenia were common laboratory abnormalities. GS-441524 and GC376 were efficient for the treatment of FIP-suspected cats.

## Supplementary Information

Supplementary Figures.

## Data Availability

All data generated or analysed during this study are included in this published article (and its Supplementary Information files).
