# Supplementary material for: A retrospective study of clinical and laboratory features and treatment on cats highly suspected of feline infectious peritonitis in Wuhan, China
Source: Sci Rep. 2021 Mar 4;11:5208. doi: 10.1038/s41598-021-84754-0 (PMC7970852; doi:10.1038/s41598-021-84754-0)

Supporting Supplementary Material for the Paper:

A retrospective study of clinical and laboratory features and treatment on cats highly suspected of feline infectious peritonitis in Wuhan, China

Yiya Yin, Ting Li, Chaohao Wang, Xiaoya Liu, Hehao Ouyang, Wanfeng Ji, Jiahao Liu, Xueyu Liao, Junyi Li & Changmin Hu

Table S1: FIP case record sheet.

Table S2: Details of 13 cats undergoing exploratory laparotomy.

Figure S1: Temperature of cats highly suspected of FIP.

Figure S2: Albumin/globulin ratio of cats highly suspected of FIP.

Figure S3: Histopathology (HE stain) and immunohistochemistry of kidney of cat no. 1.

Figure S4: Histopathology (HE stain) and immunohistochemistry of spleen of cat no. 2.

Figure S5: Histopathology (HE stain) and immunohistochemistry of liver of cat no. 3.

Figure S6: Histopathology (HE stain) and immunohistochemistry of adipose tissue of lymph node of cat no. 4.

Figure S7: Histopathology (HE stain) and immunohistochemistry of intestine of cat no. 5.

Figure S8: Histopathology (HE stain) and immunohistochemistry of lymph node of cat no. 6.

**Table S1.** FIP case record sheet

| **1.** **Basic information** | | | | | | | | | | | | | | | | | | |
| --- | --- | --- | --- | --- | --- | --- | --- | --- | --- | --- | --- | --- | --- | --- | --- | --- | --- | --- |
| **Name:** | | **Breed:** | | | | | | | **Age:** | | | **Sex:** Male □ Female □ | | | | | | |
| **Owner′s name:** | | | | | | | | | **Tel:** | | | | | | | | | |
| **Neutering status:** Intact □ | | | | | | | Neutered □ | | | | | Spayed □ | | | | | | |
| **Housing density:** Single-cat □ | | | | | | | Multi-cat □ (Quantity: ) | | | | | | | | | | | |
| **Vaccine:** No □ | | | | | | | Occasionally □ | | | | | On schedule □ | | | | | | |
| **Deworming:** No □ Occasionally □ On schedule □ Specific drug: | | | | | | | | | | | | | | | | | | |
| **Pathogen detection:** FPV-Ag( ) | | | | | | | FeLV-Ag( ) | | | | | FIV-Ag( ) Else: | | | | | | |
| **Stress:** Changing environment □ Changing food □ New pets in household □ Transport □ Else: | | | | | | | | | | | | | | | | | | |
| **2.** **Physical examination** | | | | | | | | | | | | | | | | | | |
| Date: | | |  | | |  | | | | |  | | |  | | |  | |
| Temperature: | | |  | | |  | | | | |  | | |  | | |  | |
| Respiratory rate: | | |  | | |  | | | | |  | | |  | | |  | |
| Heart rate: | | |  | | |  | | | | |  | | |  | | |  | |
| Weight: | | |  | | |  | | | | |  | | |  | | |  | |
| Dehydration assessment: | | |  | | |  | | | | |  | | |  | | |  | |
| BCS: | | |  | | |  | | | | |  | | |  | | |  | |
| **Note: ***Dehydration assessment (0-3): 0 = Severe; 1 = Moderate; 2 = Mild; 3 = No dehydration.  *BCS (body condition score, 1-5): 1 = Emaciation; 2 = Underweight; 3 = Ideal body condition; 4 = Overweight; 5 = Obesity. | | | | | | | | | | | | | | | | | | |
| **3.** **Clinical symptoms** | | | | | | | | | | | | | | | | | | |
| Date: |  | | |  |  | | |  | | Date: | | |  | |  |  | |  |
| Weight loss |  | | |  |  | | |  | | Dyspnoea | | |  | |  |  | |  |
| Lassitude |  | | |  |  | | |  | | Pleural effusion | | |  | |  |  | |  |
| Inappetence |  | | |  |  | | |  | | Ascites | | |  | |  |  | |  |
| Ocular symptoms |  | | |  |  | | |  | | Abdominal mass | | |  | |  |  | |  |
| Neurological symptoms |  | | |  |  | | |  | | Diarrhoea | | |  | |  |  | |  |
| Icterus |  | | |  |  | | |  | | Others | | |  | |  |  | |  |
| **Note:** “√” means having this symptom, “×” means not having this symptom. | | | | | | | | | | | | | | | | | | |
| **4.** **Diagnostic imaging** (Please attach the images)  *Ultrasonography:  *X-ray examination: | | | | | | | | | | | | | | | | | | |

**Table S1** (Continued)

| **5.** **Haematology** | | | | | | | | |
| --- | --- | --- | --- | --- | --- | --- | --- | --- |
| Variables | Unit | Reference interval |  | |  |  | |  |
| RBC | ×10^12^/L | 5–10 |  | |  |  | |  |
| HCT | % | 24–45 |  | |  |  | |  |
| WBC | ×10^9^/L | 5–18.9 |  | |  |  | |  |
| LYM | ×10^9^/L | 1.5–7.8 |  | |  |  | |  |
| NEU | ×10^9^/L | 2.5–12.5 |  | |  |  | |  |
| **6.** **Serum biochemistry** | | | | | | | | |
| Variables | Unit | Reference interval |  | |  |  | |  |
| TP | g/L | 57–89 |  | |  |  | |  |
| ALB | g/L | 22–40 |  | |  |  | |  |
| GLOB | g/L | 28–51 |  | |  |  | |  |
| A/G | / | / |  | |  |  | |  |
| TBIL | µmol/L | 0–15 |  | |  |  | |  |
| ALT | U/L | 12–130 |  | |  |  | |  |
| ALP | U/L | 14–111 |  | |  |  | |  |
| CRE | µmol/L | 71–212 |  | |  |  | |  |
| UREA | mmol/L | 5.7–12.9 |  | |  |  | |  |
| AMYL | U/L | 500–1500 |  | |  |  | |  |
| LIPA | U/L | 100–1400 |  | |  |  | |  |
| SAA | mg/L | 0–8 |  |  | | |  |  |
| **7. Other diagnostic methods** (Please attach the images)  ***Detection of FCoV by RT-PCR:**  Positive □ Negative □  ***Rivalta test:** Positive □ Negative □  ***Cytological examinations:**  ***Exploratory laparotomy:**  ***Histopathologic diagnosis:** | | | | | | | | |
| **8.** **Therapy** | | | | | | | | |
| **9. Supplementary notes** | | | | | | | | |
| **10. Outcome:** Cured □ Died naturally □ Euthanasia □  Symptoms before death: | | | | | | | | |
| **11.** **Doctor signature:** **Date:** | | | | | | | | |

**Table S2.** Details of 13 cats undergoing exploratory laparotomy

| **No.** | **Breed** | **Sex** | **Age (months)** | **Neutered** | **Housing density** | **Stressor** | **Clinical symptom** | **CBC** | **Serum biochemistry** | **Rivalta** | **RT-PCR** | **Exploratory laparotomy** | **Histopathology** | **IHC** | **Outcome** |
| --- | --- | --- | --- | --- | --- | --- | --- | --- | --- | --- | --- | --- | --- | --- | --- |
| 1 | BSH | F | 4 | N | MC | ND | Weight loss  Lassitude  Inappetence Fever  AM  Dyspnoea  PE  Ascites | WBC↑  LYM↓  NEU↑ | GLOB↑  A/G = 0.537  ALT↑  CRE↓ | + | + | 1. Enlarged mesenteric lymph nodes  2. Surface of the mesentery was scattered with miliary pink nodules of different sizes  3. Surface of the kidney was scattered with miliary white nodules  4. Surface of the spleen was covered with an opalescent fibrinous film, and the edge became blunt and round | 1. Moderate multifocal pyogranulomatous lymphadenitis  2. Moderate focal pyogranulomatous interstitial nephritis  3. Marked multifocal to coalescing pyogranulomatous steatitis | +++ | Euthanasia |
| 2 | ESH | F | 24 | Y | MC | ND | Weight loss  Lassitude  Inappetence  Fever  Dyspnoea  Ascites | RBC↓  HCT↓  LYM↓ | GLOB↑  A/G = 0.443  CRE↓  UREA↓ | + | - | 1. Large number of miliary pale yellow nodules were scattered on the intestinal surface  2.Intestine adhered to the abdominal wall  3. Kidney was mildly enlarged and structurally unclear  4. Liver was earthy yellow, covered with a thin creamy yellow fibrinous film  5. Spleen was covered with a thick creamy yellow fibrinous film  6. Surface of the diaphragm was scattered with miliary white nodules  7. Middle lobe of the lung turned white | 1. Mild lymphoplasmahistiocytic hepatitis  2. Moderate to marked serofibrinatous and pyogranulomatous perisplenitis  3. Moderate to marked focal pyogranulomatous serositis of the small intestine | +++ | Euthanasia |
| 3 | BSH | F | 7 | N | ND | ND | Weight loss  Lassitude  Inappetence  AM  Diarrhoea  Ascites | LYM↑  NEU↓ | ALB↓  A/G = 0.311  TBIL↑  CRE↓  UREA↓ | + | + | 1. Enlarged mesenteric lymph nodes  2. Accretive adhesion mass about 9 cm long from the end of the ileum to the anterior segment of the colon; after being cut open, the intestinal wall was found to be significantly thickened with oedema  3. Surface of the liver was scattered with miliary white nodules | 1. Moderate coalescing pyogranulomatous lymphadenitis  2. Severe coalescing, transmural pyogranulomatous enteritis of the small and large intestine  3. Multifocal moderate pyogranulomatous hepatitis | +++ | Euthanasia |
| 4 | Crossbred | F | 1 | N | SC | CE | Weight loss  Lassitude  Inappetence  Ascites  NS | RBC↓  HCT↓  WBC↑  LYM↓ | ALB↓  A/G = 0.467  CRE↓  UREA↓  SAA↑ | + | + | 1. Enlarged mesenteric lymph nodes  2. Kidney structure was not clear  3. Liver was earthy yellow and enlarged  4. Spleen was covered with a thin white fibrinous film | 1. Mild to moderate lymphohistiocytic perivascular inflammation of the adipose tissue  2. Mild purulent hepatitis | + | Euthanasia |
| 5 | Crossbred | F | 24 | N | ND | ND | Ascites | WBC↑  LYM↑  NEU↓ | GLOB↑  A/G = 0.483 | + | + | 1. Surface of intestine was scattered with miliary pale yellow nodules  2. Ascites was very viscous | Marked chronical multifocal to coalescing pyogranulomatous colitis | +++ | Euthanasia |
| 6 | BSH | M | 14 | Y | ND | ND | AM  Ascites | ND | ND | + | - | 1. Enlarged mesenteric lymph nodes  2. Surface of the intestine and mesentery was scattered with miliary pink nodules  3. Surface of the liver was covered with slightly raised white nodules of different sizes | 1. Chronical severe pyogranulomatous and fibrosing inflammation  2. Chronical marked multifocal to coalescing pyogranulomatous hepatitis | +++ | Euthanasia |

**Table S2** (Continued)

| **No.** | **Breed** | **Sex** | **Age (months)** | **Neutered** | **Housing density** | **Stressor** | **Clinical symptom** | **CBC** | **Serum biochemistry** | **Rivalta** | **RT-PCR** | **Exploratory laparotomy** | **Histopathology** | **IHC** | **Outcome** |
| --- | --- | --- | --- | --- | --- | --- | --- | --- | --- | --- | --- | --- | --- | --- | --- |
| 7 | Ragdoll | F | 14 | N | MC | ND | Weight loss  Lassitude  Inappetence  Icterus  AM  Ascites | RBC↓  WBC↑  NEU↓ | TP↑  GLOB↑  A/G = 0.250  TBIL↑  AGP↑ | + | + | 1. Enlarged mesenteric lymph nodes  2. White nodules of soybean-like size were scattered on the surface of the liver | Multifocal pyogranulomatous hepatitis | ND | Euthanasia |
| 8 | BSH | M | 6 | N | MC | NP | Weight loss  Lassitude  Inappetence  Fever  AM  Diarrhoea Ascites | HCT↓  WBC↓  LYM↓ | GLOB↑  A/G = 0.519  ALP↓  UREA↓ | + | + | 1. Surface of mesentery was scattered with miliary pink nodules  2. Spleen was covered with a thin white fibrinous film, and the spleen parenchyma showed multifocal white lesions | ND | ND | Euthanasia |
| 9 | Crossbred | F | 24 | N | MC | CE | Inappetence AM  Ascites | HCT↓  WBC↑  LYM↓ | TP↑  GLOB↑  A/G = 0.256  TBIL↑  CRE↓  UREA↓ AMYL↑ | ND | + | 1. Enlarged mesenteric lymph nodes  2. Surface of mesentery was scattered with miliary yellow nodules | ND | ND | Cured by GS-441524 |
| 10 | BSH | M | 8 | N | MC | NP | Weight loss  Lassitude  Inappetence  Icterus  Ascites | LYM↓ | ALB↓  A/G = 0.314 | + | + | 1. Enlarged kidney  2. Enlarged liver  3. Liver and spleen were covered with a thin white fibrinous film  4. Right lung was scattered with haemorrhagic spots of different sizes, with part of the lobe turning white | 1. Serofibrinatous and pyogranulomatous perisplenitis  2. Multifocal pyogranulomatous hepatitis | ND | Euthanasia |
| 11 | Crossbred | F | 24 | N | ND | ND | Ascites | All normal | A/G = 0.767  AMYL↑ | + | + | 1. Surface of the spleen was dotted with small white nodules  2. Left kidney was extremely enlarged and thin with urinary retention, and the right kidney was normal | ND | ND | Cured by GC376 |
| 12 | Crossbred | F | 98 | Y | MC | ND | Weight loss  Lassitude  Inappetence  AM  Dyspnoea  PE | ND | ND | ND | ND | 1. Enlarged mesenteric lymph nodes  2. Multiple coin-sized white nodules appeared on the surface of the liver | ND | ND | Euthanasia |
| 13 | BSH | F | 24 | Y | MC | ND | Lassitude  Inappetence  Ascites  OS | ND | ND | ND | ND | Liver was earthy yellow, and the surface was scattered with miliary white nodules | Multifocal pyogranulomatous hepatitis | ND | Euthanasia |

**Notes**: CBC: Complete blood count; IHC: Immunohistochemistry; ESH: Exotic Shorthair; BSH: British Shorthair; F: Female; M: Male; N: No; Y: Yes; MC: Multi-cat; SC: Single-cat; ND: Not determined; CE: Changing environment; NP: New pets in the household; AM: Abdominal mass; PE: Pleural effusion; OS: Ocular symptoms; NS: Neurological symptoms; “↑”: Increased; “↓”: Decreased; “+++”: Strongly positive; “+”: Positive; “-”: Negative.

**Figure S1.** Temperature of cats highly suspected of FIP.


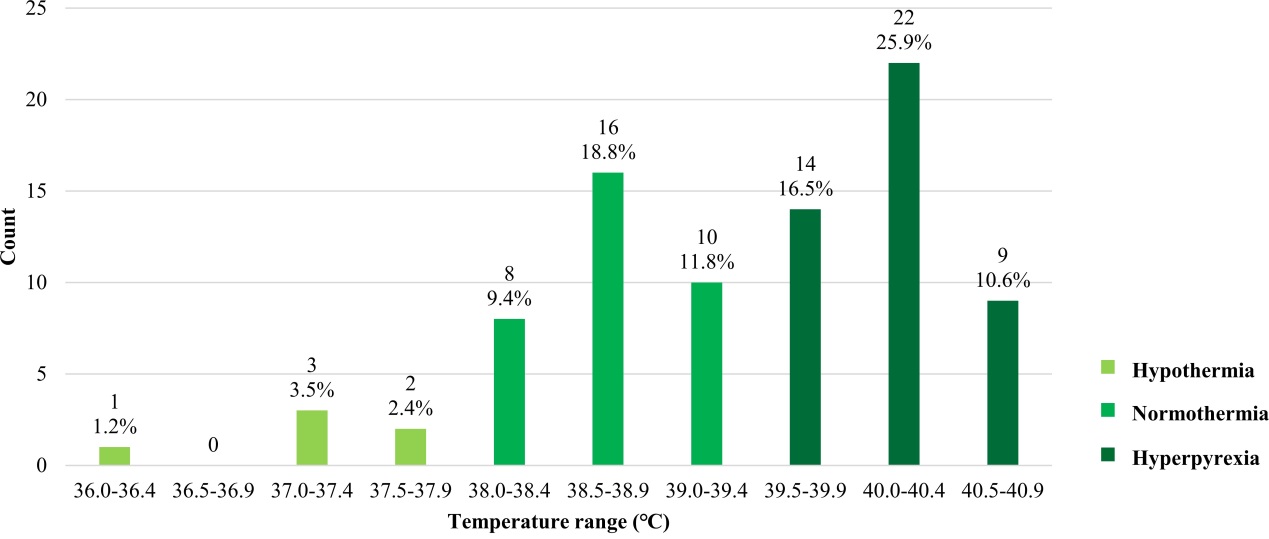


**Figure S2.** Albumin/globulin ratio of cats highly suspected of FIP.


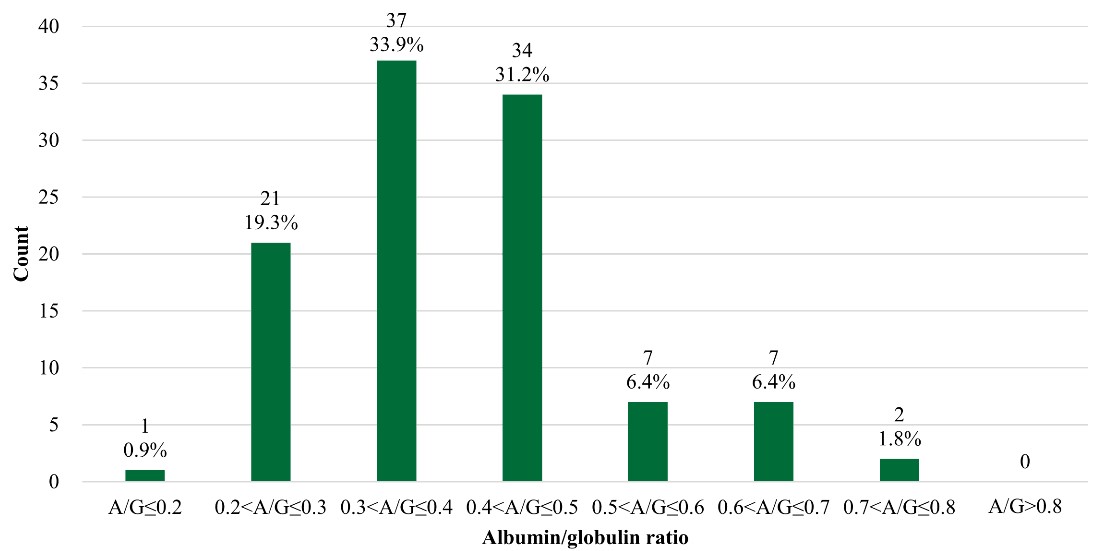


**Figure S3:** Histopathology (HE stain) and immunohistochemistry of kidney of cat no. 1.


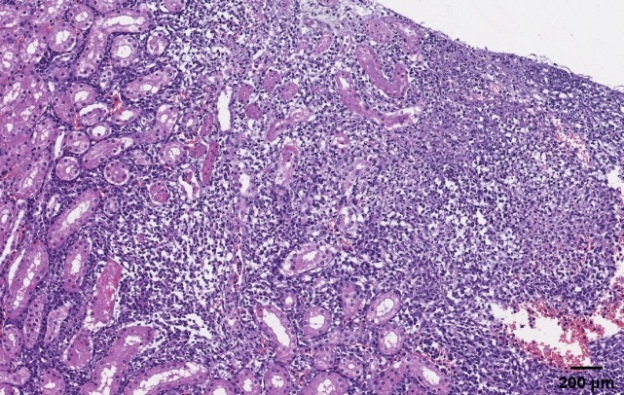

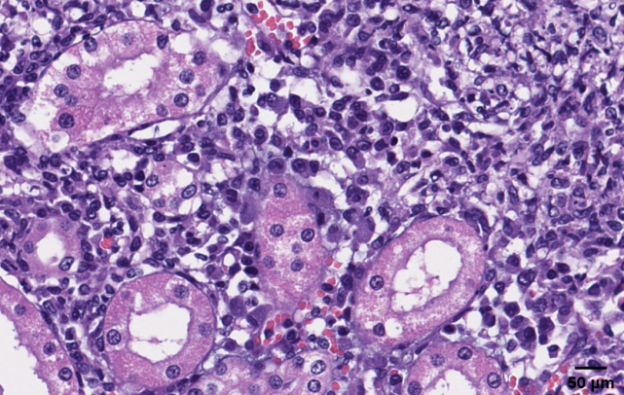


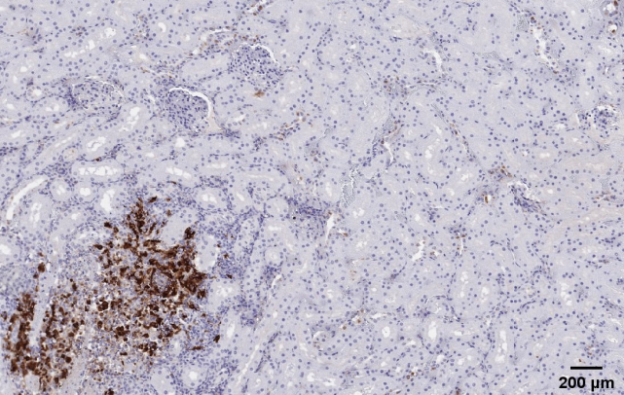

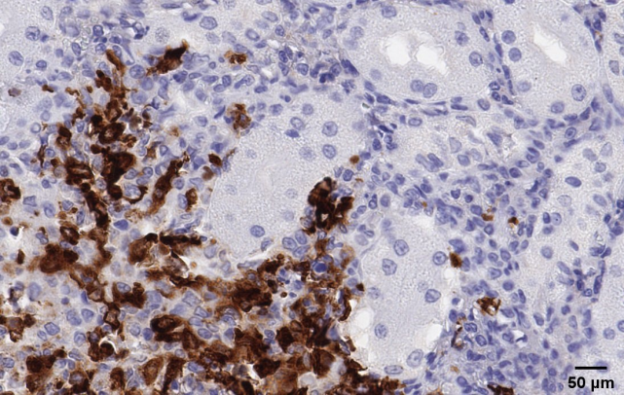


**Figure S4.** Histopathology (HE stain) and immunohistochemistry of spleen of cat no. 2.


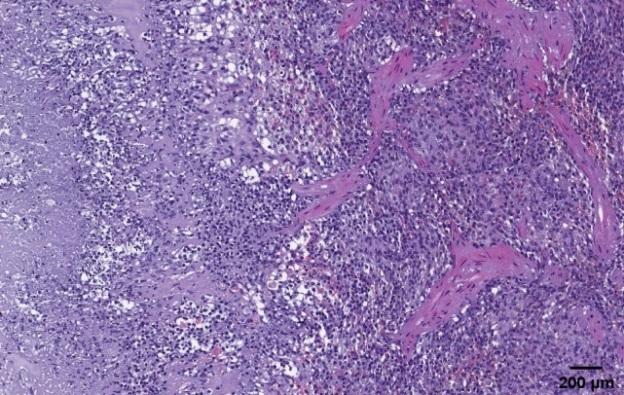

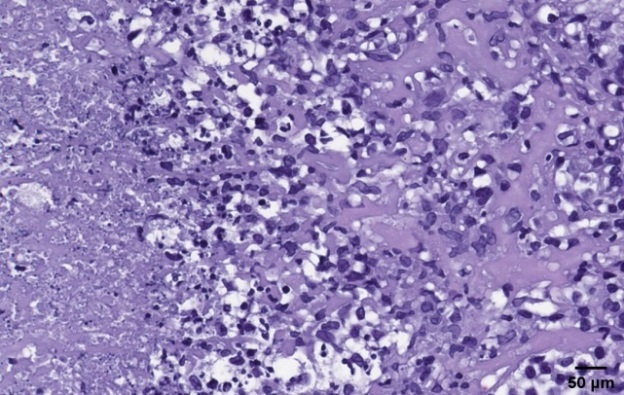

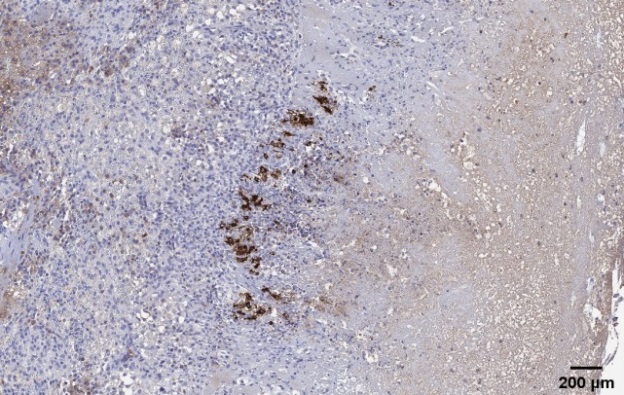

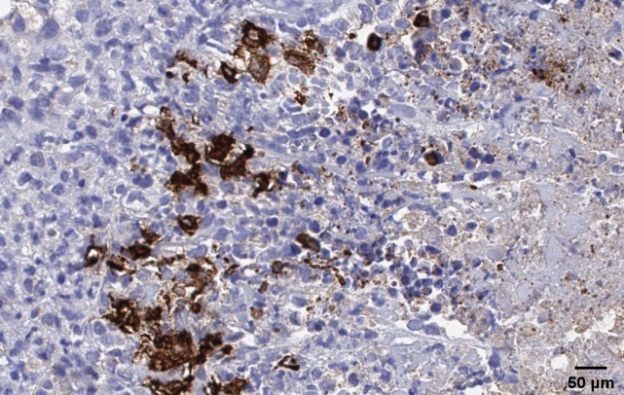


**Figure S5.** Histopathology (HE stain) and immunohistochemistry of liver of cat no. 3.

**
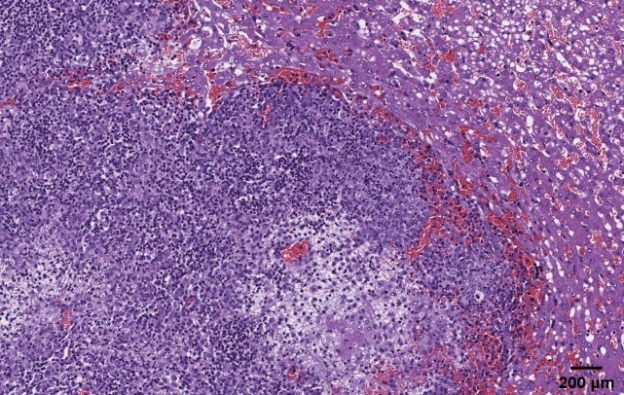

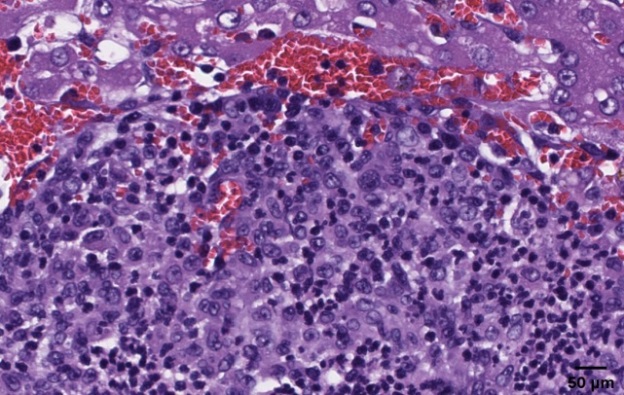

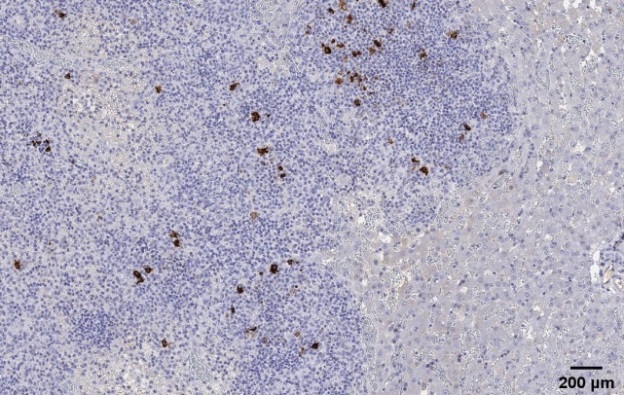

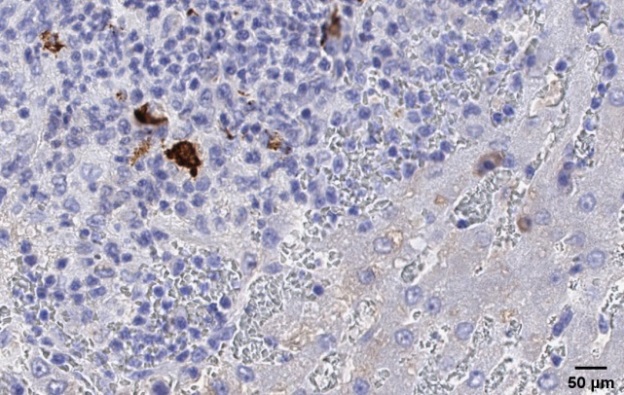
**

**Figure S6.** Histopathology (HE stain) and immunohistochemistry of adipose tissue of lymph node of cat no. 4.

**
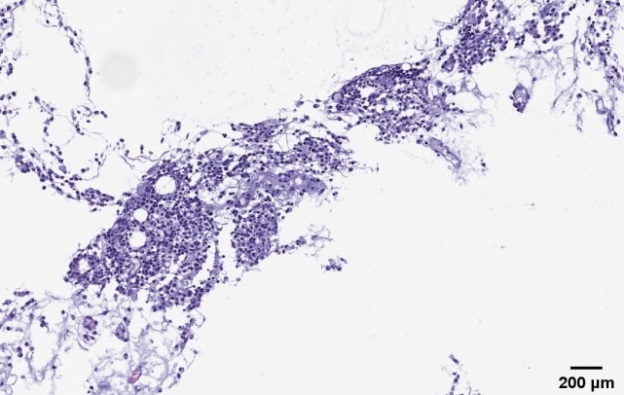

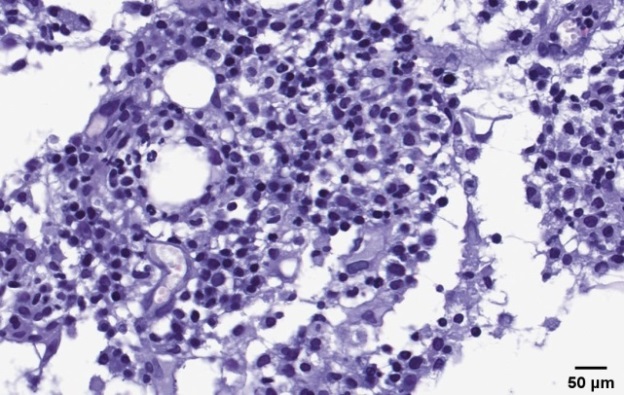

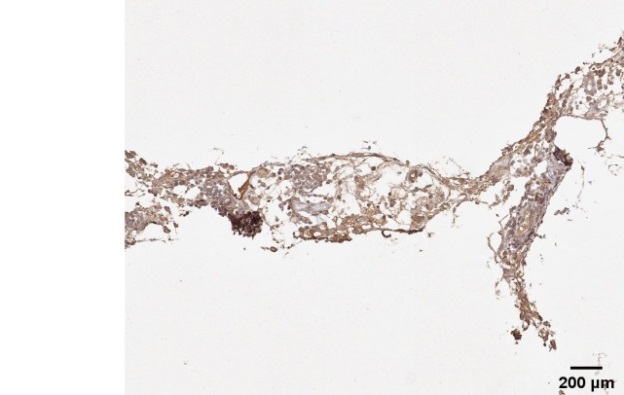

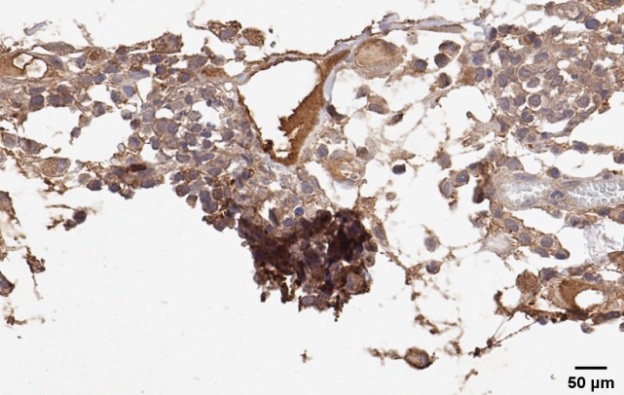
**

**Figure S7.** Histopathology (HE stain) and immunohistochemistry of intestine of cat no. 5.


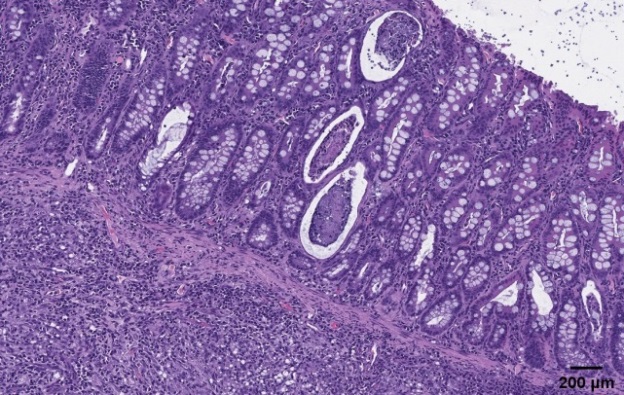

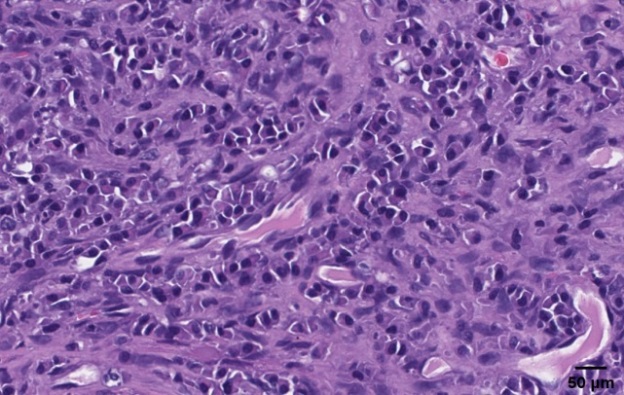

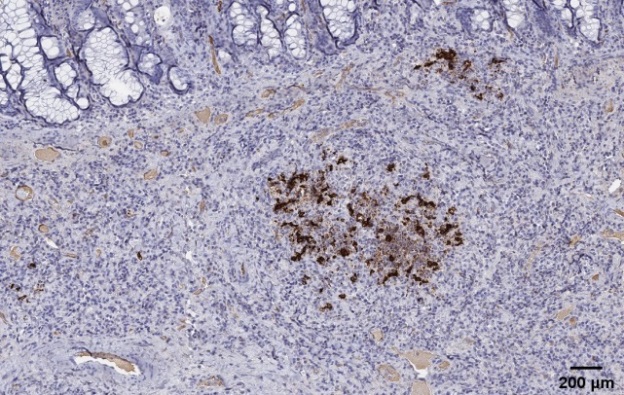

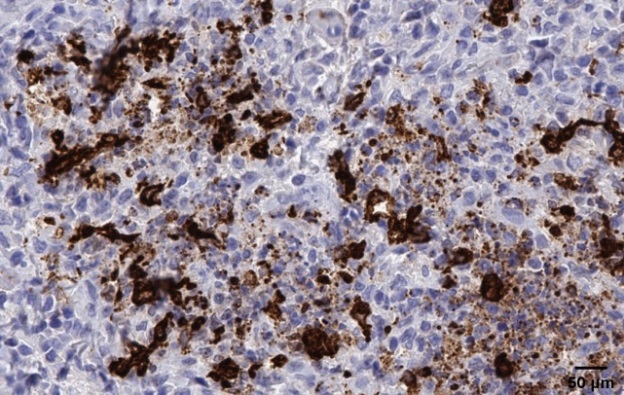


**Figure S8.** Histopathology (HE stain) and immunohistochemistry of lymph node of cat no. 6.


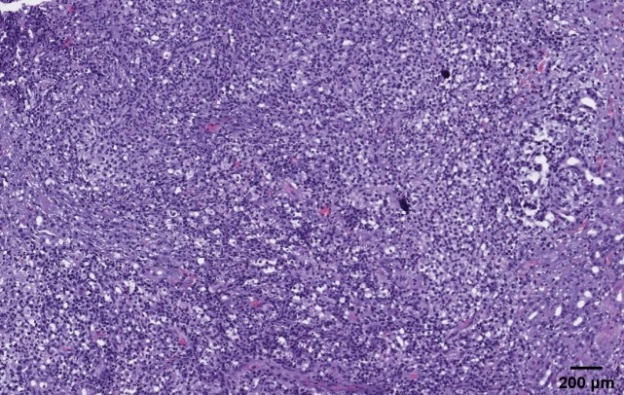

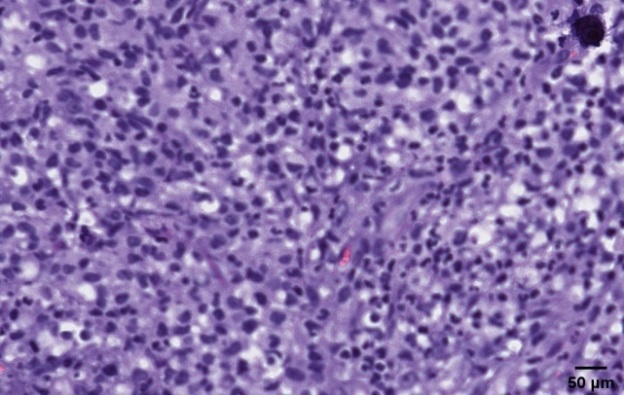

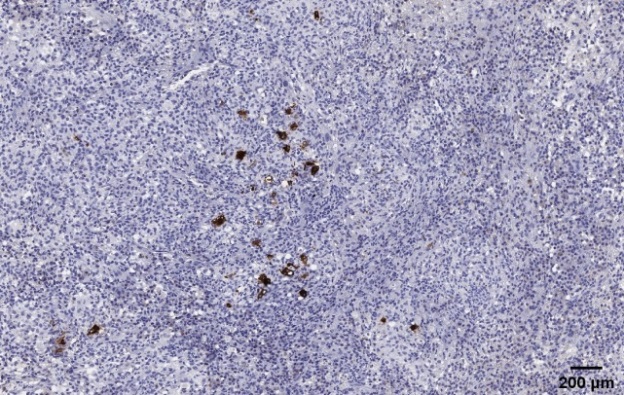

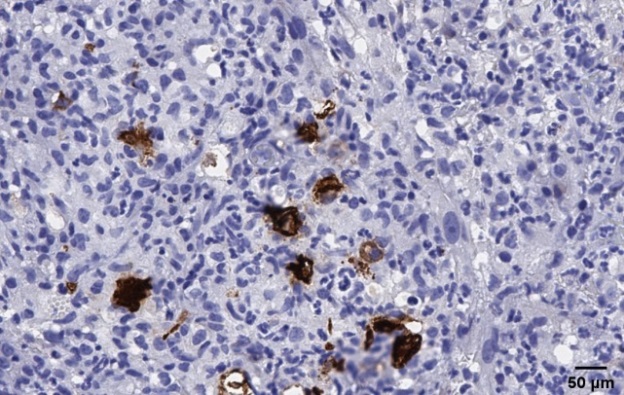

Supplement: Supplementary file 1 — Supplementary Figures. [file 41598_2021_84754_MOESM1_ESM.docx]
